# Identification of N-Terminally Truncated Derivatives of Insulin Analogs Formed in Pharmaceutical Formulations

**DOI:** 10.1007/s11095-018-2426-1

**Published:** 2018-05-16

**Authors:** Joanna Zielińska, Jacek Stadnik, Anna Bierczyńska-Krzysik, Dorota Stadnik

**Affiliations:** Institute of Biotechnology and Antibiotics (IBA), Starościńska 5, 02-516 Warsaw, Poland

**Keywords:** insulin, insulin analogs, insulin impurities, peptide mass fingerprinting, truncation

## Abstract

**Purpose:**

Isolation and identification of unknown impurities of recombinant insulin lispro (produced at IBA) formed during accelerated stability testing of pharmaceutical solutions. For comparative purposes also commercially available formulations of recombinant human insulin (Humulin S®; Lilly), recombinant insulin lispro (Humalog®; Lilly), recombinant insulin aspart (NovoRapid® Penfill®; Novo Nordisk), recombinant insulin detemir (Levemir®; Novo Nordisk) and recombinant insulin glargine (Lantus®; Sanofi-Aventis) were analyzed.

**Methods:**

The impurities of insulin analogs were isolated by RP-HPLC and identified with peptide mass fingerprinting using MALDI-TOF/TOF mass spectrometry.

**Results:**

The identified derivatives were N-terminally truncated insulin analog impurities of decreased molecular mass of 119, 147 and 377 Da related to the original protein. The modifications resulting in a mass decrease were detected at the N-terminus of B chains of insulin lispro, insulin aspart, human insulin, insulin glargine, insulin detemir in all tested formulations. To our knowledge it is the first time that these impurities are reported.

**Conclusions:**

The following derivatives formed by truncation of the B chain in insulin analogs were identified in pharmaceutical formulations: desPhe^B1^-N-formyl-Val^B2^ derivative, desPhe^B1^ derivative, pyroGlu^B4^ derivative.

**Electronic supplementary material:**

The online version of this article (10.1007/s11095-018-2426-1) contains supplementary material, which is available to authorized users.

## Introduction

Recombinant human insulin and its analogs are commonly used to treat diabetes mellitus. The binding of the protein’s monomeric form to the insulin receptor (IR) enables regulation of the blood glucose level and influences the lipid and protein metabolism [1]. Nowadays, these therapeutics are produced by recombinant DNA technology [2] and are commercially available as various formulations, both soluble solutions and suspensions with protamine [3]. A detailed description of these formulation can be found elsewhere [4–6]. In distinction to small molecule drugs, biopharmaceuticals represent large, heterogeneous and complex class of medicines [7]. Their physical and chemical stability determining the drug’s efficacy and safety remains a great challenge in protein development. In order to obtain the drug approval by regulatory authorities, recombinant therapeutics are strictly monitored to detect, characterize and finally eliminate or considerably limit undesirable by-products. These include derivatives formed during expression, purification and long-term storage of the biopharmaceuticals which are further evaluated in terms of toxicity and biological activity [8, 9]. Moreover, despite high structural similarity to a parent protein, chemical modifications induced during oxidation and the perturbation of the secondary structure often result in enhanced immunogenicity of the second generation products [10, 11]. A variety of other reported modifications includes deamidation, transamidation, racemization, oxidation, glycation, cross-links formation and disulphide scrambling [12]. The rate of derivatives formation strongly depends on the pH, temperature and ionic strength of the aqueous medium [13]. Also the ratio of individual components may vary depending on conditions. Most often, deamidation of insulin at residues Asn A21, Asn B3, and Gln B4 is described [14–16]. The reaction proceeds in aqueous solution under both acidic and neutral conditions. As a result of storage in acidic pH, insulin preparations deamidate primarily in AsnA21 forming AspA21 [17]. In neutral conditions deamidation at AsnB3 occurs. It leads to the formation of AspB3 and isoAspB3 products [14]. By racemization, D-aspartyl derivatives may be produced [18–20]. Moreover, Brange characterized a hydrolysis product resulting from the cleavage of the peptide bond between ThrA8 and SerA9, occurring in Zn^2+^-rich solutions, containing rhombohedral crystals [21]. Furthermore, the author reports formation of covalent insulin dimers during storage of pharmaceutical preparations. In solutions containing protamine analogous, insulin-protamine products are formed [22]. The above processes, as well as formation of high molecular weight transformation products are regarded relatively slow in comparison to deamidation [22, 23]. Other insulin derivatives determined in insulin aspart include isoAsp^B28^ and desPhe^B1^-N-oxalyl-Val^B2^ [16]. The latter one, resulting in 75 Da deficit, was also identified in human insulin at neutral conditions [24]. Here we present identification of novel desPhe^B1^-, desPhe^B1^-N-formyl-Val^B2^- and pyroGlu^B4^ insulin derivatives formed spontaneously in pharmaceutical solutions.

## Materials and Methods

### Chemicals

All chemicals were of analytical reagent grade. Hydrochloric acid 35–38%, acetonitrile, sodium hydroxide were purchased from Avantor (Center Valley, PA, USA). Sodium perchlorate, phosphoric acid 85%, trifluoroacetic acid (TFA), ammonium carbonate were purchased from Merck (Darmstadt, Germany). Triethylamine, dithiothreitol (DTT), iodoacetamide (IAA), formic acid, HEPES were purchased from Sigma-Aldrich (Munich, Germany). Endoproteinase Glu-C Protease *S. aureus* V8 and pepsin were purchased from MP Biomedicals (Santa Ana, California, USA). Pharmaceutical formulations: recombinant human insulin (Humulin S®) and recombinant insulin lispro (Humalog®) were from Eli Lilly (Indianapolis, IN, USA), recombinant insulin lispro (Insulin KP drug product) was from IBA (Warsaw, Poland), recombinant insulin aspart (NovoRapid® Penfill®) and recombinant insulin detemir (Levemir®) were from Novo Nordisk (Bagsværd, Denmark), recombinant insulin glargine (Lantus®) was from Sanofi-Aventis (Frankfurt, Germany).

### Isolation of the Truncated Derivatives

Prior to isolation all pharmaceutical formulations were incubated one month in the stability chambers at +37°C/65% RH in the original cartridges. All the above-mentioned products differed in aging time when incubation was started. The truncated derivatives were isolated from the pharmaceutical formulations by repetitive reversed phase chromatography (RP-HPLC) using 2695 Alliance system (Waters, Milford, USA) equipped with 2489 U*V*/VIS detector (214 nm). Data acquisition and processing were conducted using Empower software. 0.1 ml of the pharmaceutical formulations was injected on two Supelcosil LC-18-dB 150 mm × 4.6 mm, 3 μm columns connected in series. The separation was carried out at 40°C with gradient elution at 1 mL/min, run time 60 min: (0–35 min) isocratic elution at A/B = 46/54; (35–55 min) linear change to A/B = 10/90; (55–60 min) isocratic elution at A/B = 10/90. Eluent A was 14 mM sodium perchlorate, 3.7 mM triethylamine, 4.7 mM phosphate buffer and 5.5% (vol/vol) acetonitrile, pH 2.3. Eluent B was 6.5 mM sodium perchlorate, 1.7 mM triethylamine, 2.2 mM phosphate buffer and 50.3% (vol/vol) acetonitrile, pH 2.3. The peaks of the derivatives with relative retention times (RRT) of 0.59; 0.78; 0.81 were collected with Fraction Collector III (Waters Milford, USA). This procedure was repeated several times to obtain sufficient amount of the derivatives from each pharmaceutical formulation. All fractions of the derivatives were pooled and evaporated to dryness using Concentrator Plus vacuum centrifuge (Eppendorf, Hamburg, Germany) and kept at −20°C until use.

### Isolation of the B Chain

The derivatives were dissolved in 0.5 ml of 0.1 M ammonium bicarbonate solution pH 8.0 and mixed with 10 μl of 0.05 M DTT. The mixture was incubated for 40 min at 50°C. Then 20 μl of 0.1 M IAA was added and the mixture was incubated for 1 h at 25°C in darkness. Then the HPLC system (Waters Alliance 2695, Milford, USA) equipped with a Zorbax SB-C18 1.8, 50 mm × 4.6 mm column (Agilent, Santa Clara, CA, USA), was employed to separate and isolate the B chain. The separation was carried out at 40°C with a linear gradient elution from 10 to 50% eluent B in 20 min at a flow rate 1 ml/min. Eluent A was 0.1% TFA and eluent B was 0.1% TFA with 90% ACN (both vol/vol). The peak of the B chain was collected with Fraction Collector III (Waters, Milford, USA) and eluent was evaporated as mentioned above.

### Enzymatic Digestion of the B Chain of Derivatives

The B chains of the derivatives were dissolved in 0.1 ml of 0.1 M HEPES buffer pH 7.5 and mixed with endoproteinase Glu-C at a concentration 10 μg/ml with a mass ratio of enzyme/polypeptide chain = 1/50. The mixture was incubated for 1 h at 37°C. Digestion was stopped by acidifying to pH 2 with 10% formic acid. The mixture was incubated for 15 min at 25°C. The samples were kept at −20°C until use.

### MALDI-TOF/TOF

Mass spectra were acquired in a positive-ion reflector mode with the use of a 4800 Plus MALDI-TOF/TOF Analyzer (Applied Biosystems, Framingham, USA). Alpha-cyano-4-hydroxycinnamic acid (CHCA) from Sigma-Aldrich (Munich, Germany), dissolved in 50:50 water/acetonitrile (J.T. Baker, Deventer, The Netherlands) with 0.1% TFA – final concentration (Sigma-Aldrich, Munich, Germany), was exploited as a MALDI matrix. External calibration was achieved with a 4700 proteomics analyzer calibration mixture provided by Applied Biosystems. Samples were spotted onto a 384 Opti-TOF MALDI plate and analyzed. Data Explorer Software, Version 4.9 was applied to process acquired spectra. Mascot Distiller Software (version 2.5.1.0, Matrix Science) was employed to predict fragment ions from given peptide sequences and overlay them on the acquired MS/MS spectra.

## Results and Discussion

**Fig. 1:**
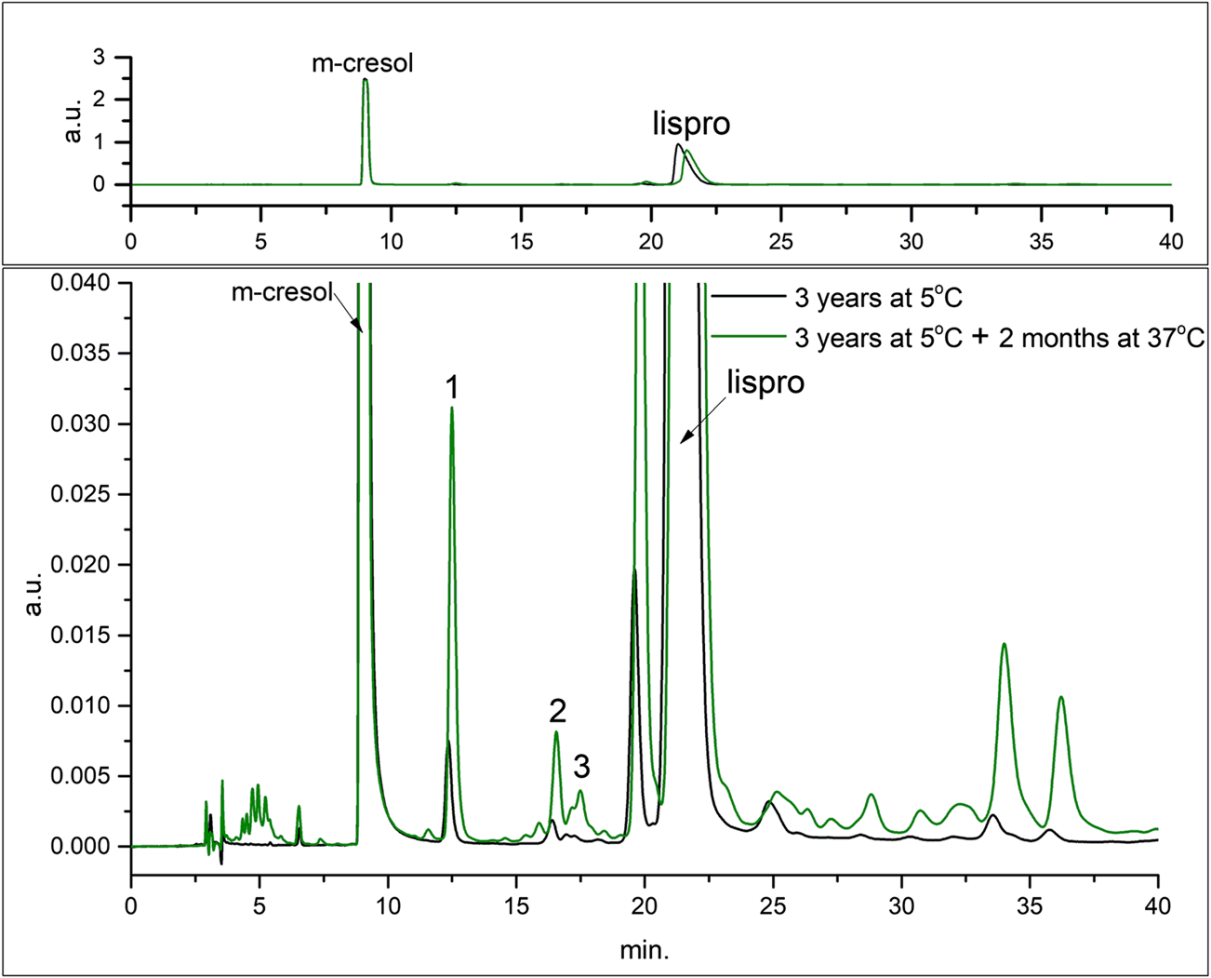
Chromatogram of Insulin lispro drug product (IBA) during stability studies: after 3 years at +5°C (black line) and after 3 years at +5°C + two months at +37°C (green line). Two main peaks in this chromatogram are m-cresol (antimicrobial preservative) and insulin lispro (API). Small peaks arising from the baseline (seen in enlargement in the bottom panel) are “related proteins”.

The studied insulin lispro formulations were manufactured at Institute of Biotechnology and Antibiotics with the implementation of recombinant DNA technology. An integral part of formulation development was stability testing which provides data to estimate the drug’s shelf-life and storage conditions. Long-term and accelerated stability studies were performed for the insulin lispro drug product. The degradation of insulin lispro was monitored by RP-HPLC method and exemplary chromatograms obtained as a result of the analysis performed are shown in Fig. 1.

**Fig. 2:**
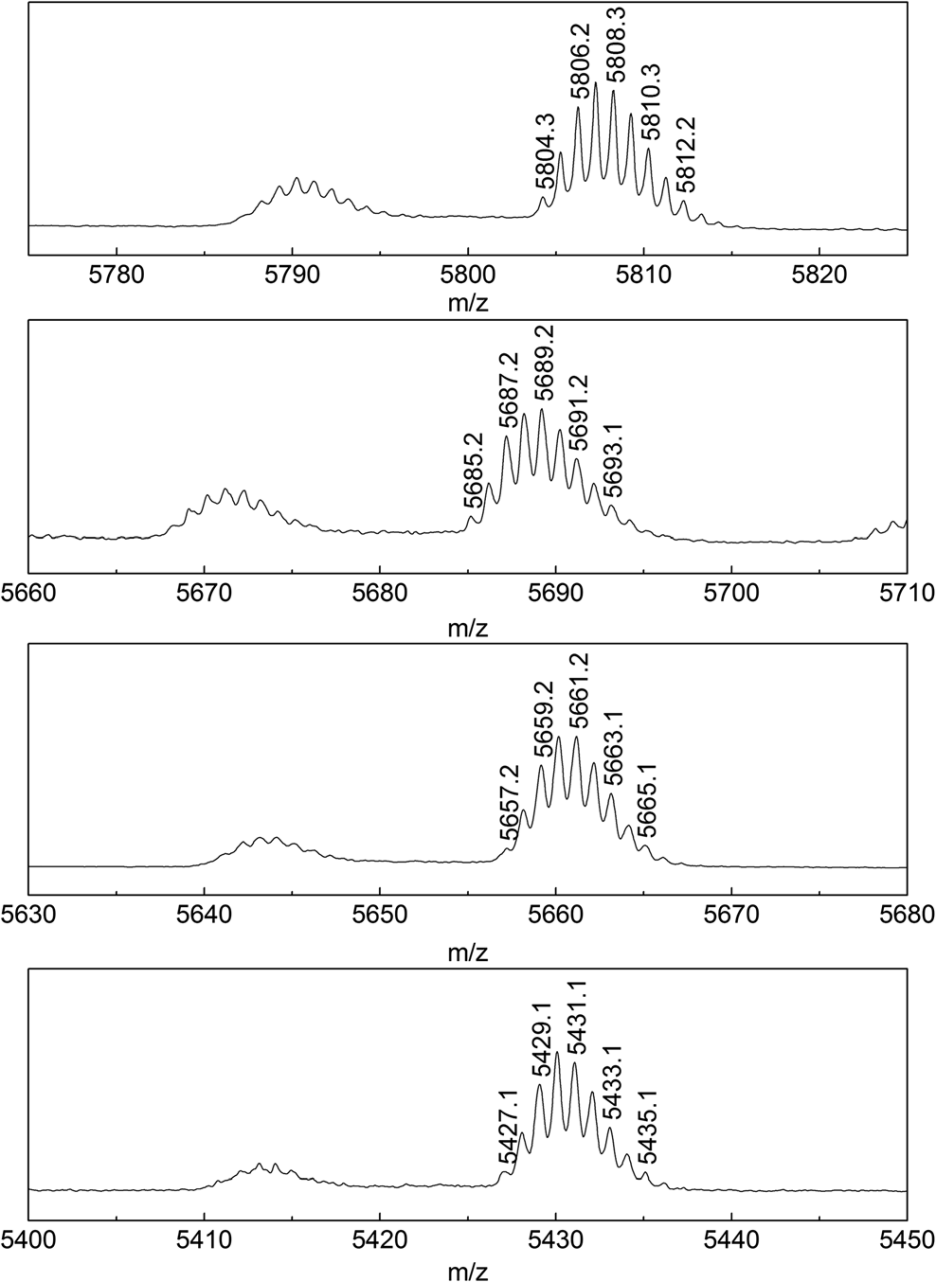
Mass spectra of insulin lispro and its derivatives 1, 2, 3 (from top to bottom).

**Fig. 3:**
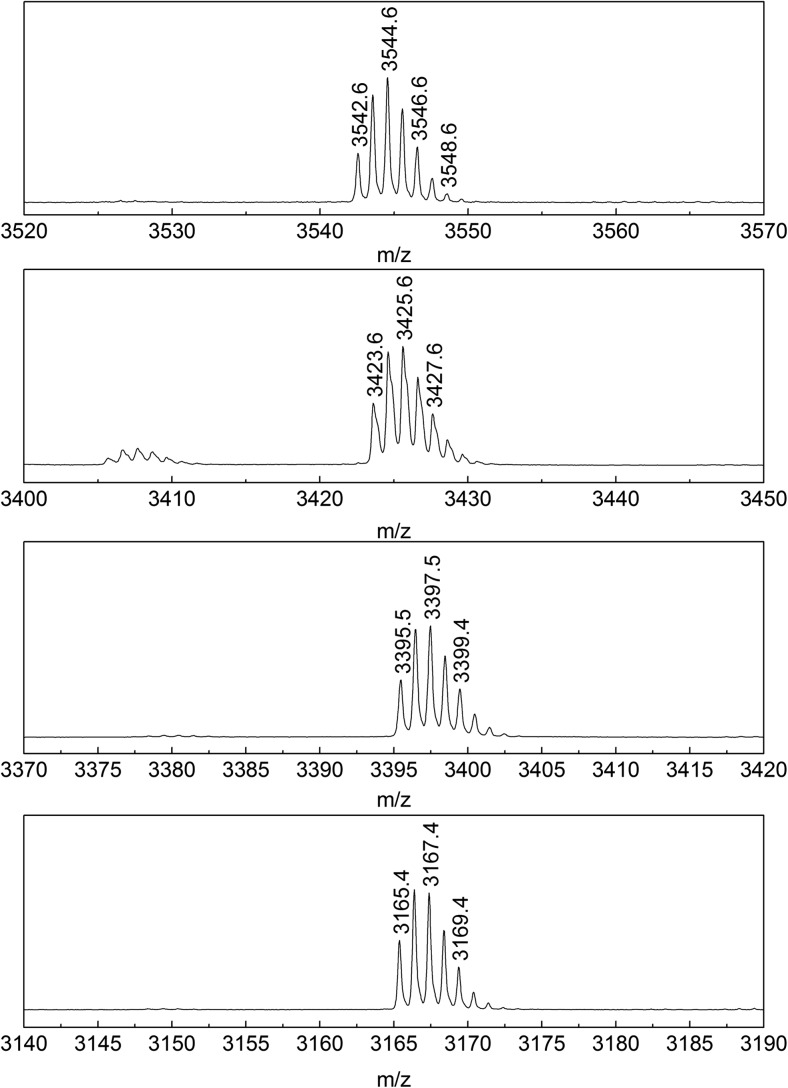
Mass spectra of the B chain of insulin lispro and its derivatives 1, 2, 3 (from top to bottom).

Three derivatives eluting before insulin lispro, labeled as 1, 2, 3 in Fig. 1, were isolated and characterized with MALDI-TOF/TOF mass spectrometry. The recorded spectra (Fig. 2) revealed the derivatives of decreased molecular mass of 119, 147 and 377 Da related to the insulin lispro. The formation of protein impurities with mass reduced by 75 Da was observed in aspart, human, beef and pork insulin formulations [11]. These were desPhe^B1^-oxalyl-Val^B2^ derivatives with the modified Phe residue at the N-terminal of the B chain. Based on this research we predicted that isolated derivatives 1, 2 and 3 were the products of the further truncation of amino acids from the N-terminal of the B chain. Indeed, the first experiment involving reduction and alkylation of isolated fractions and MALDI-TOF MS analysis has shown that the observed mass decrease is due to the changes at the B chain amino acid sequence. The molecular mass of the B chain of derivative 1, 2, 3 was smaller by respectively 119, 147 and 377 Da than the molecular mass of the B chain of insulin lispro as shown in Fig. 3.

**Fig. 4:**
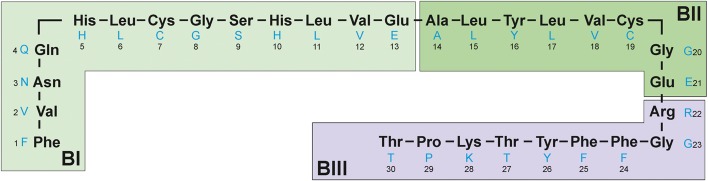
Peptide fragments of the B chain of insulin lispro after digestion with protease V8.

To determine whether the modifications take place at the N-terminus of B chains, the B chains of all derivatives under investigation and insulin lispro were subjected to digestion with protease V8 followed by MALDI-TOF/TOF MS analysis. The complete digestion of the B chain of insulin lispro with protease V8 results in three fragments: BI (B1-B13) BII (B14-B21), BIII (B22-B30) (Fig. 4).

**Fig. 5:**
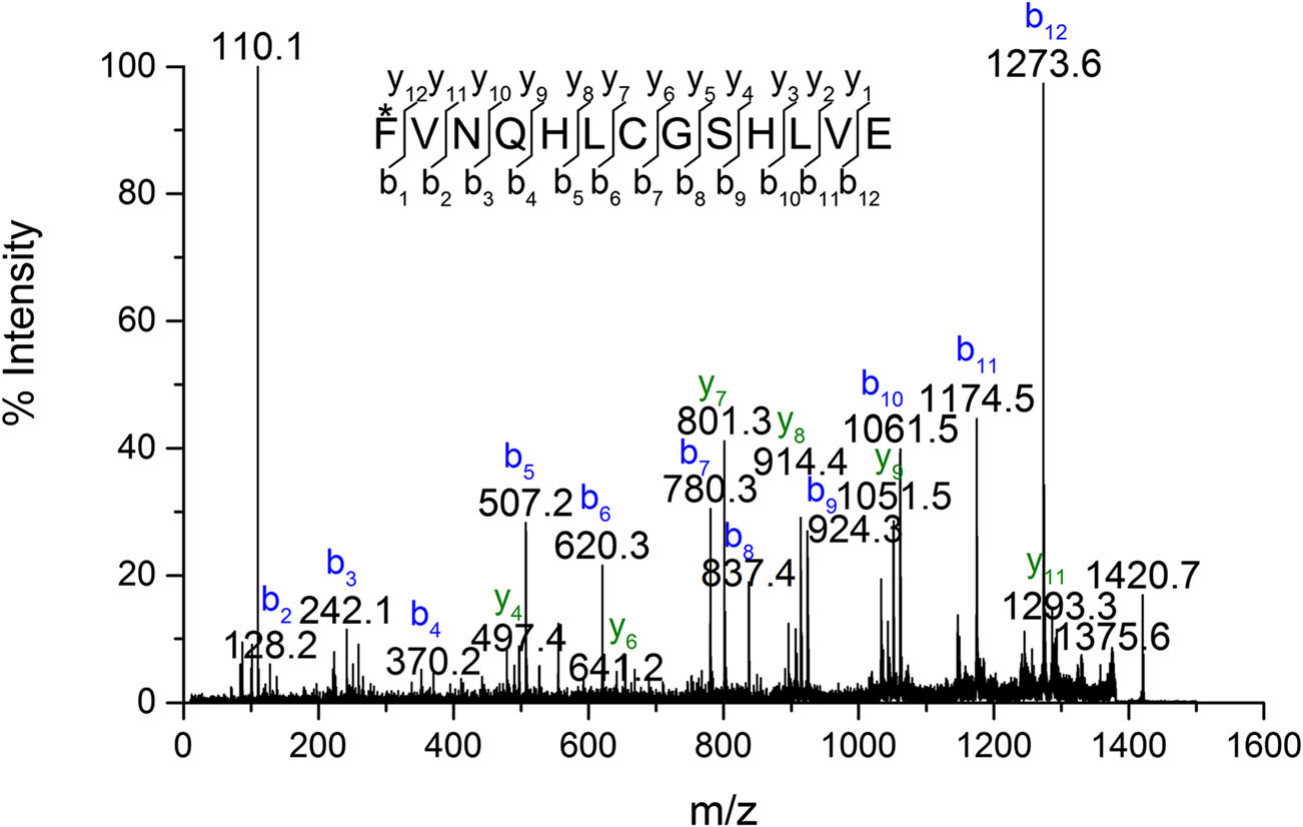
MS/MS spectrum of peptide B1-B13 from desPhe^B1^-N-formyl-Val^B2^ derivative.

The BI peptide of insulin lispro has a monoisotopic mass of 1539.7 Da. In the derivatives’ digests, ions at m/z 1420.7 (≈1539.7–119), 1392.7 (≈1539.7–147), 1162.5 (≈1539.7–360-17) were detected and assigned to the truncated BI peptides of the investigated derivatives. For further structural elucidation, these ions were subjected to MS/MS sequencing. Figure 5 shows the fragmentation spectrum of m/z 1420.7, which was assigned to the BI peptide of derivative 1. All y-type ions seen in the spectrum were in agreement with theoretical, unmodified sequence VNQHLCGSHLVE, whereas b-type ions were shifted by 119 units in comparison to the theoretical values for the BI peptide in insulin lispro. Based on these data, it was concluded that only the N-terminal amino acid residue in the BI peptide could be modified resulting in a mass decrease by 119 Da giving the sequence as follows: F_mod_VNQHLCGSHLVE. Upon closer inspection of the MS/MS spectrum it can be noticed that precursor ion peak at m/z 1420.7 is accompanied by the cognate peak at m/z 1375.5 with a loss of 44 Da corresponding to the elimination of the formylamido group (–NHCHO) from the N-terminal of the BI peptide. Taking into account the data presented and analogy to previously identified desPhe^B1^-N-oxalyl-Val^B2^ insulin, derivative 1 was recognized as desPhe^B1^-N-formyl-Val^B2^ insulin lispro. This derivative does not have the N-terminal NH_2_- group at the B chain which was also confirmed by Edman degradation (see Fig. S-1 in supplementary materials).

**Fig. 6:**
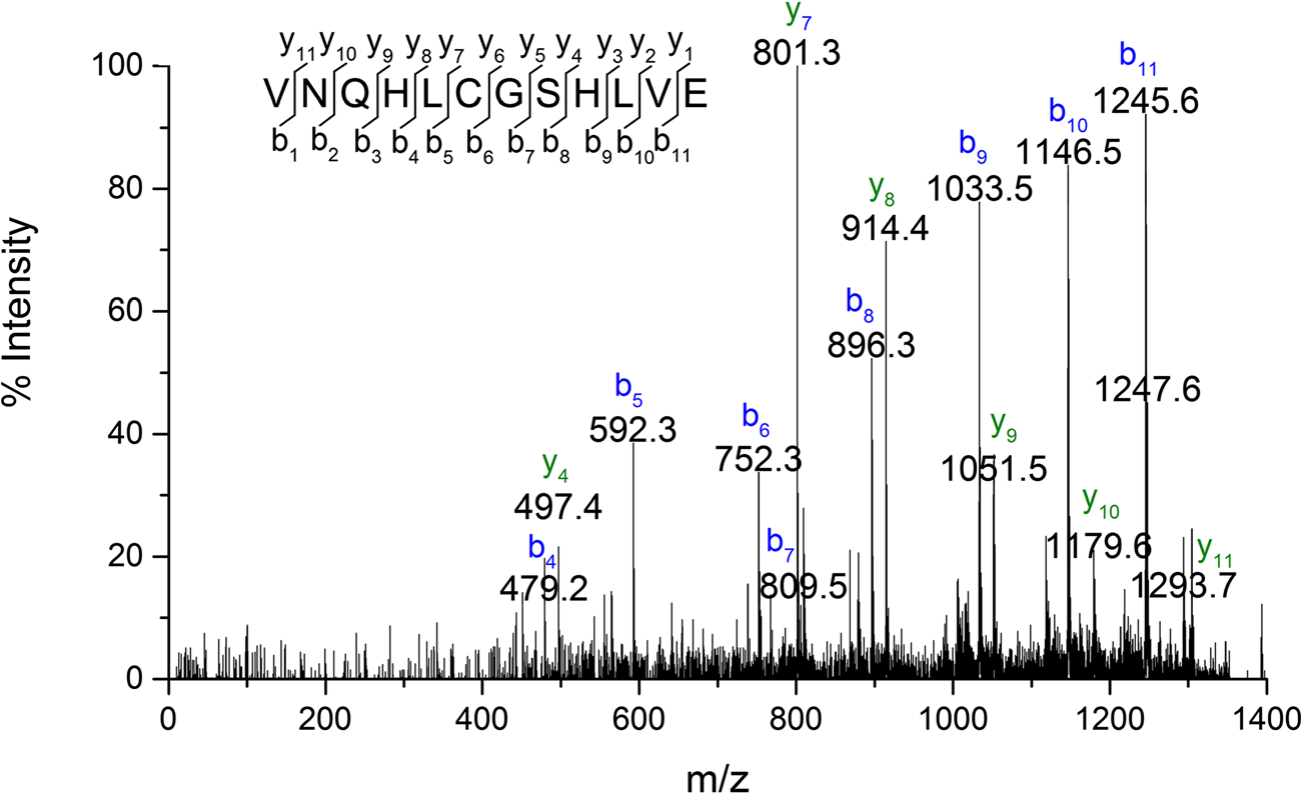
MS/MS spectrum of peptide B2-B13 from desPheB1 derivative.

In case of derivative 2, the observed mass (m/z 1392.7) of fragment BI differs by 147 Da from the mass of BI of insulin lispro (m/z 1539.7) which coincides with the absence of the N-terminal phenylalanine residue. The MS/MS spectrum of the ion at m/z 1392.7 (Fig. 6) corresponds to the truncated B1 peptide sequence VNQHLCGSHLVE. Based on these data derivative 2 was identified as desPheB1 insulin lispro.

**Fig. 7:**
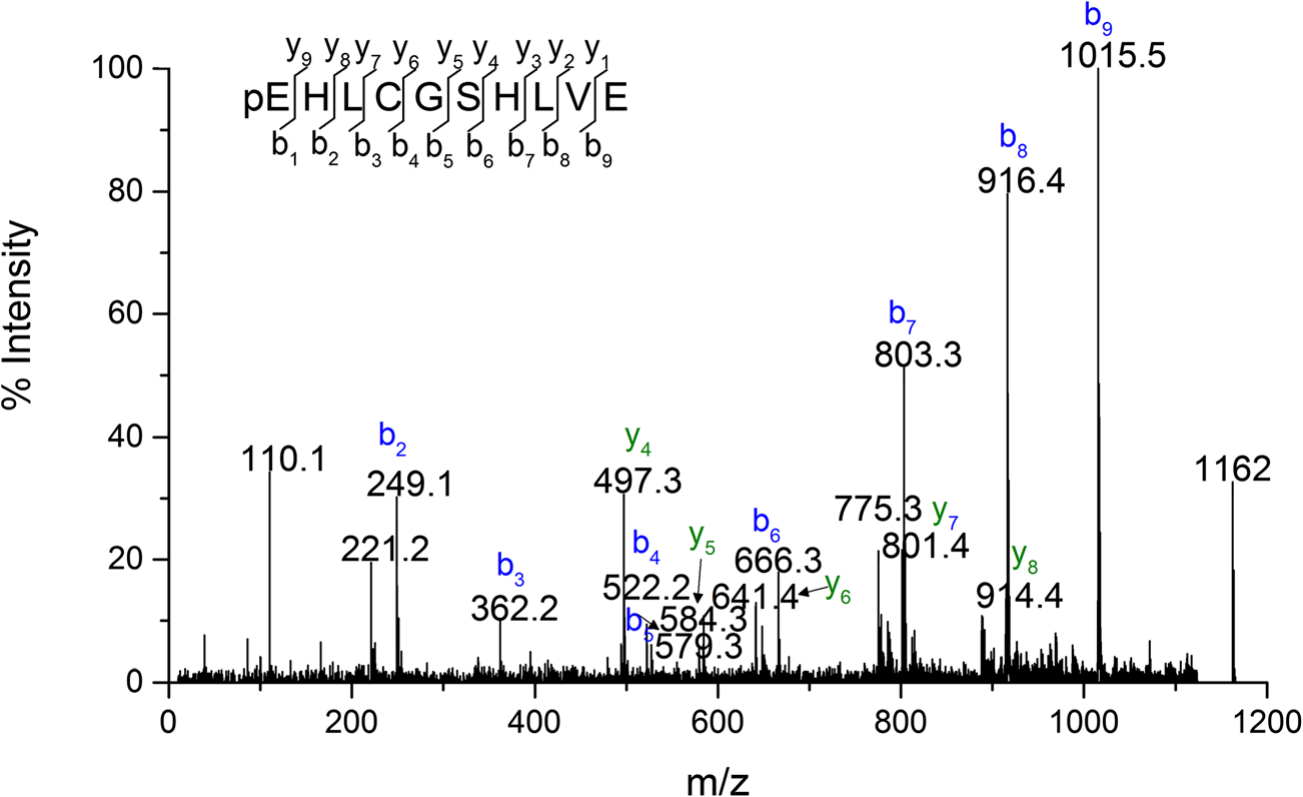
MS/MS spectrum of peptide B4-B13 from pyroGluB4 derivative.

**Fig. 8:**
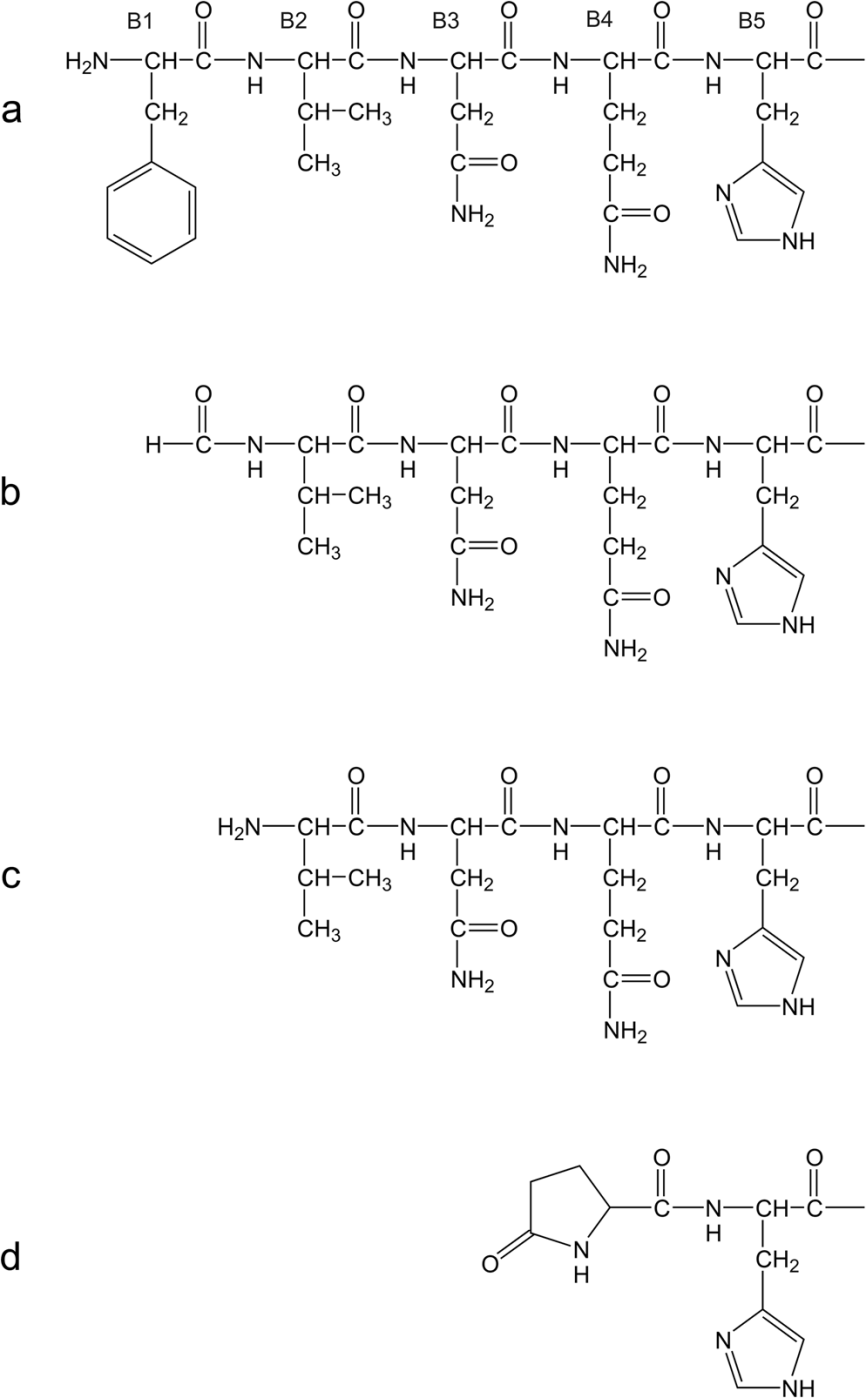
Scheme of the N-terminal residues from B chain of (**a**) insulin lispro (the first five residues of the B chain are shown for clarity), (**b**) desPhe^B1^-N-formyl-Val^B2^ derivative, (**c**) desPhe^B1^ derivative, (**d**) pyroGlu^B4^ derivative.

Referring to the above result, we assumed that derivative 3 was also a product of truncation of N-terminal residues from the B chain. The mass difference of 377 Da was indicative of the loss of three amino acid residues FVN (≈360 Da) and a moiety of 17 Da. Once the tripeptide FVN is absent from the sequence FVNQHLCGSHLVE, the next amino acid is glutamine (Q) which can readily cyclize to form pyroglutamate. The process is accompanied by the loss of NH_3_ and a mass decrease of 17 Da [25]. In the MS/MS spectrum of BI fragment of derivative 3 (Fig. 7), b ions corresponding to the sequence with the 17 Da loss from QHLCGSHLVE were detected beginning at b2, whereas y-series of ions were found to be unchanged. Therefore derivative 3 was identified as pyroGluB4 insulin lispro, where the −377 Da modification was assigned to the N-terminus of the B chain as a loss of FVN residues (−360 Da) and ammonia (−17 Da). The structures of pyroGlu^B4^ derivative and the remaining identified derivatives are presented in Fig. 8.

All identified derivatives are products of truncation of N-terminal residues from the B chain. What is interesting, the truncated derivatives were detected also in the formulations of other studied analogs (see Fig. S-2 – Fig. S-7 in supplementary materials) irrespective of the type of insulin (human insulin, insulin lispro, insulin aspart, insulin glargine, insulin detemir) used as the active pharmaceutical ingredient and pH of the formulation (e.g. all tested formulations have a pH 7 whereas glargine (Lantus®) has a pH of ~4).

The truncation process is relatively slow in insulin pharmaceutical solutions. Elevated levels of truncated derivatives were detected in formulations subjected to incubation at +37°C. The content of truncated insulin lispro derivatives in the insulin pharmaceutical formulations does not exceed EP specification limits (0.50%; any other impurity as specified in European Pharmacopoeia, monograph 01/2008:2085) during the long term stability studies at +5°C. The mechanism of truncation of proteins in insulin formulations is not fully known, however a presence of reducing agents and metal ions may play a role. The autocatalytic cleavage of peptide bond A8-A9 was observed in crystalline insulin suspensions containing surplus zinc ions in addition to of those structurally bound to insulin [21]. R. Torosantucci *et al.* reported formation of insulin fragments during oxidation of insulin in the oxidative Cu(II)/ascorbate system [26]. Copper (II), as redox active metal ions, were also reported to be responsible for non-enzymatic fragmentation of an IgG1 monoclonal antibody [27]. Although excipients used in all tested formulations are not reducing substances themselves they can be contaminated with trace amounts of such compounds. The formation of desPhe^B1^-N-formyl-Val^B2^ derivative can proceed by similar pathway as was proposed for desPhe^B1^-N-oxalyl-Val^B2^ derivative [16]. The transformation involves a Maillard reaction between insulin analogs and the reducing substances and subsequent hydrolytic degradation. Both derivatives desPhe^B1^ and pyroGlu^B4^ are products of non-enzymatic hydrolysis of insulin lispro. The latter also requires a cyclization reaction. Non-enzymatic hydrolysis of proteins and conversion of N-terminal Glu to pGlu was observed in a presence of reducing agents and metal ions [28, 29]. Therefore, we anticipate that, these factors are the most suspicious in formation of identified truncated derivatives.

## Conclusions

In 2002 M. U. Jars *et al.* published a paper on derivatives of insulin aspart [11]. One of the described derivatives was desPhe^B1^-N-oxalyl-Val^B2^ insulin with truncated N-terminus of B chain. In our study, we present identification of consecutive derivatives of decreasing molecular mass which are formed by further truncation of this chain in insulin lispro: desPhe^B1^-N-formyl-Val^B2^ derivative, desPhe^B1^ derivative, pyroGlu^B4^ derivative. These derivatives were isolated from pharmaceutical formulations of insulin lispro produced at IBA. They were detected in the formulations of other analyzed analogs irrespective of the type of analog that was the active pharmaceutical ingredient in the formulation.

## Electronic supplementary material

ESM 1 (DOCX 876 kb)

## Abbreviations

DTT: Dithiothreitol
EP: European Pharmacopoeia
HEPES: 2-[4-(2-hydroxyethyl)piperazin-1-yl]ethanesulfonic acid
IAA: Iodoacetamide
IBA: Institute of Biotechnology and Antibiotics, Warsaw, Poland
TFA: Trifluoroacetic acid
