# Supplementary material for: Identification of N-Terminally Truncated Derivatives of Insulin Analogs Formed in Pharmaceutical Formulations
Source: Pharm Res. 2018 May 16;35(7):143. doi: 10.1007/s11095-018-2426-1 (PMC5956049; doi:10.1007/s11095-018-2426-1)
Supplement: Supplementary file 1 — (DOCX 876 kb) [file 11095_2018_2426_MOESM1_ESM.docx]

SUPPLEMENTARY INFORMATION TO

**Identification of N-terminally truncated derivatives of insulin analogs formed in pharmaceutical formulations**

Joanna Zielińska, Jacek Stadnik, Anna Bierczyńska-Krzysik, Dorota Stadnik*

Institute of Biotechnology and Antibiotics, Starościńska 5, 02-516 Warsaw, Poland,

***Corresponding Author**

Dorota Stadnik

Department of Chemical Analysis

Institute of Biotechnology and Antibiotics

Starościńska 5

02-516 Warsaw

Poland

Phone: +48 22 3786155

E-mail: stadnikd@iba.waw.pl

**Table S-1 Composition of insulin formulations**

| Name of the medicinal product (manufacturer) | API | API concentration | Inactive ingredients | Packaging |
| --- | --- | --- | --- | --- |
| Humulin S®  (Eli Lilly) | human insulin | 3.5 mg/ml  100 IU/ml | m-cresol: 2.7 mg/ml  glycerol: 16 mg/ml  Zn: 30 µg/ml  water  Hydrochloric acid and/or sodium hydroxide may be added to adjust pH. Humulin S® has a pH of 6.9-7.8. | cartridges |
| Humalog®  (Eli Lilly) | insulin lispro | 3.5 mg/ml  100 U/ml | sodium phosphate, dibasic: 1.88 mg/ml  m-cresol: 3.15 mg/ml  phenol: trace  glycerol: 16 mg/ml  Zn: 19.7 µg/ml  water  Hydrochloric acid and/or sodium hydroxide may be added to adjust pH. Humalog® has a pH of 7.0-7.8. | cartridges |
| Insulin KP drug product (IBA) | insulin lispro | 3.5 mg/ml  100 U/ml | sodium phosphate, dibasic: 1.88 mg/ml  m-cresol: 3.15 mg/ml  phenol: trace  glycerol: 16 mg/ml  Zn: 19.7 µg/ml  water  Hydrochloric acid and/or sodium hydroxide may be added to adjust pH. Humalog has a pH of 7.0-7.8. | cartridges |
| Lantus®  (Sanofi-Aventis) | insulin glargine | 3.64 mg/ml  100 U/ml | m-cresol: 2.7 mg/ml  glycerol: 16 mg/ml  Zn: 30 µg/ml  water  The pH is adjusted by addition of aqueous solutions of hydrochloric acid and sodium hydroxide. Lantus® has a pH of approximately 4. | cartridges |
| NovoRapid®Penfill®  (Novo Nordisk) | insulin aspart | 3.5 mg/ml  100 U/ml | disodium phosphate dihydrate: 1.25 mg/ml  m-cresol: 1.72 mg/ml  phenol: 1.5 mg/ml  glycerol: 16 mg/ml  Zn: 19.6 µg/ml  NaCl: 0.58 mg/ml  water  Hydrochloric acid and/or sodium hydroxide may be added to adjust pH. NovoRapid® has a pH of 7.2-7.6. | cartridges |
| Levemir®  (Novo Nordisk) | insulin detemir | 14.2 mg/ml  100 U/ml | disodium phosphate dihydrate: 0.89 mg/ml  m-cresol: 2.06 mg/ml  phenol: 1.8 mg.ml  glycerol: 16 mg/ml  Zn: 65.4 µg/ml  NaCl: 1.17 mg/ml  water  Hydrochloric acid and/or sodium hydroxide may be added to adjust pH. Levemir® has a pH of approximately 7.4. | cartridges |

**N-terminal sequencing**

Determination of the N-terminal sequence of insulin lispro and desPheB1-N-formyl-ValB2 derivative by Edman degradation was performed on a Procise 491 (Applied Biosystems, Forster City, USA) automatic protein sequencer. Before analyses the instrument was calibrated using a commercial PTH proteinaceous amino acid standard mixture (Wako, Osaka, Japan). The sequencing was done using polypeptide chains absorbed on TFA-treated glass fiber disks (Wako, Osaka, Japan) coated by polybrene (Sigma, St. Louis, USA).

Insulin lispro used as a reference and desPheB1-N-formyl-ValB2 derivative are two chain proteins. Both chains are connected with disulphide bridges, therefore two amino acids are released during each cycle of the Edman degradation. As can be seen in Figure S-1, two amino acids were detected during the first two cycles of lispro sequencing. In case of desPhe^B1^-N-formyl-Val^B2^ derivative, only one amino acid was released from the A chain indicating that the B chain was blocked.


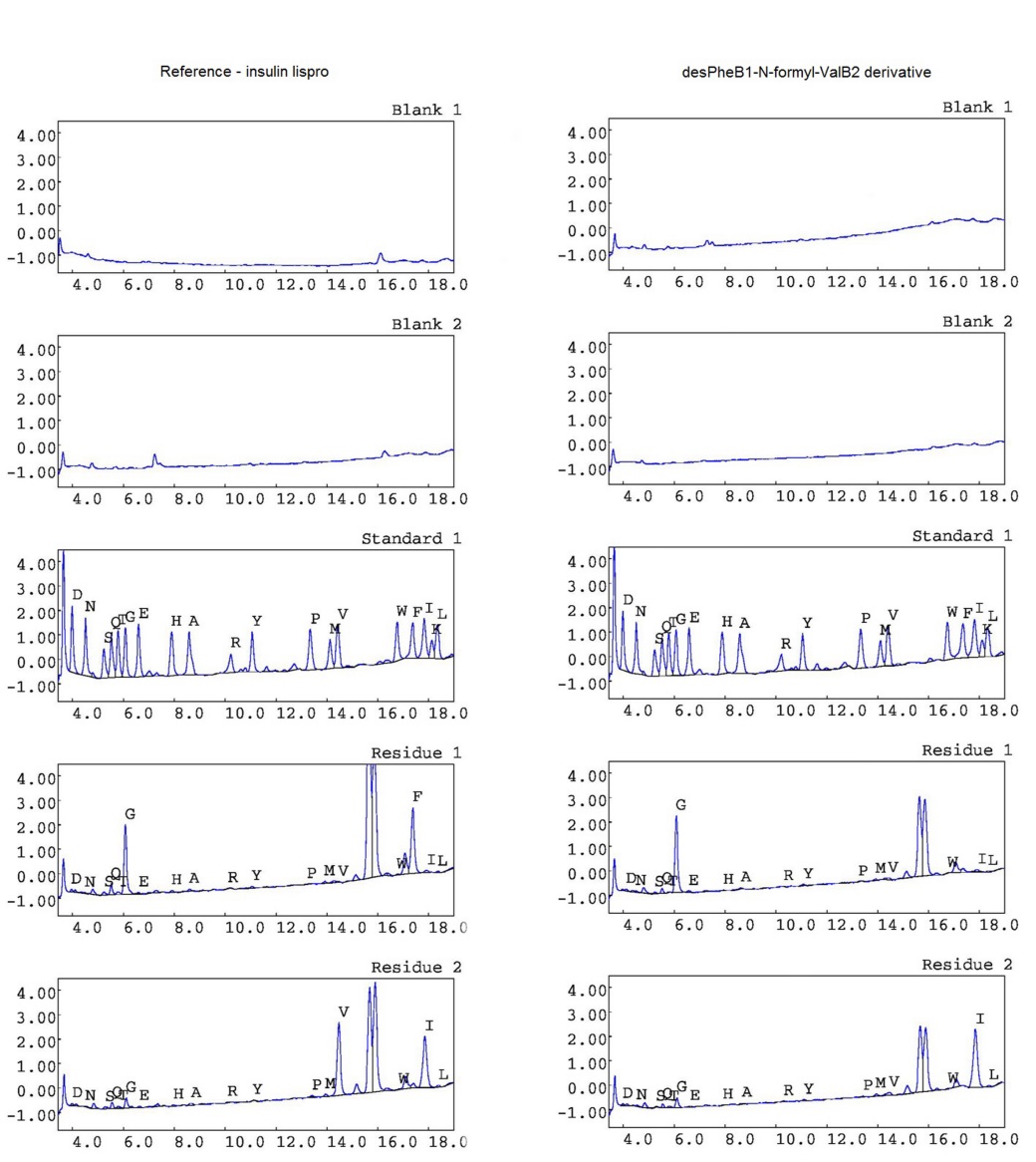


Figure S-1. HPLC chromatograms of the PTH amino acid residues detected during sequencing by automated Edman degradation. Left panel (from top to bottom): blank1, blank 2, amino acid standard, residue 1 in A and B chain of insulin lispro, residue 2 in A and B chain of insulin lispro. Right panel (from top to bottom): blank1, blank 2, amino acid standard, residue 1 in A and B chain of desPhe^B1^-N-formyl-Val^B2^ derivative, residue 2 in A and B chain of desPhe^B1^-N-formyl-Val^B2^ derivative.





Figure S-2. Mass spectra of desPhe^B1^ insulin aspart.





Figure S-3. Mass spectra of pyroGlu^B4^ insulin aspart.





Figure S-4. Mass spectra of desPhe^B1^-N-formyl-Val^B2^ insulin glargine.





Figure S-5. Mass spectra of desPhe^B1^ insulin glargine.





Figure S-6. Mass spectra of desPhe^B1^-N-formyl-Val^B2^ insulin detemir.





Figure S-7. Mass spectra of desPhe^B1^ insulin detemir.
